# Design and Integrated Analysis of a Flexible Support Microstructure with a Honeycomb Sandwich for the Optical Window of a Hypersonic Remote Sensor

**DOI:** 10.3390/s21175919

**Published:** 2021-09-02

**Authors:** Mingqiang Zhang, Yaobin Li, Yalin Ding, Jianjun Sun, Jing Li

**Affiliations:** 1 Changchun Institute of Optics, Fine Mechanics and Physics, Chinese Academy of Sciences, Changchun 130033, China; zhangmingqiang18@mails.ucas.edu.cn (M.Z.); liyaobin@ciomp.ac.cn (Y.L.); sunjianjun@ciomp.ac.cn (J.S.); lijing187@mails.ucas.edu.cn (J.L.); 2 University of Chinese Academy of Sciences, Beijing 100049, China

**Keywords:** hypersonic remote sensor, honeycomb composite structure, imaging quality, flexible support, optical window, integration analysis

## Abstract

In order to reduce the influence of the optical window on the image quality of a hypersonic visible light optical remote sensor, we propose a method of adding a double-layer semicircular honeycomb core microstructure with flexible support of a high temperature elastic alloy between a window glass and a frame to reduce the influence of complex thermal stress on the surface accuracy of the optical window. An equivalent model of a semicircular honeycomb structure was established, the elastic parameters of the semicircular honeycomb sandwich microstructure were derived by an analytical method, and a numerical verification and finite element simulation were carried out. The results show that the equivalent model is in good agreement with the detailed model. The optical-mechanical-thermal integrated simulation analysis of the optical window assembly with flexible supporting microstructure proves that the semicircular honeycomb sandwich flexible supporting structure has a positive effect on stress attenuation of the window glass and ensures the wave aberration accuracy of the transmitted optical path difference of the optical window (PV < 0.665 λ, RMS < 0.156 λ, λ = 632.8 nm). Combined with the actual optical system, the optical performance of the window assembly under the flexible support structure is verified. The simulation results show that the spatial frequency of the modulation transfer function (MTF) of the optical system after focusing is not less than 0.58 in the range of 0–63 cycle/mm and the relative decline of MTF is not more than 0.01, which meet the imaging requirements of a remote sensor. The study results show that the proposed metal-based double-layer semicircular honeycomb sandwich flexible support microstructure ensures the imaging quality of the optical window under ultra-high temperature conditions.

## 1. Introduction

In recent years, aerial remote sensors have been applied more and more widely in military and civil fields, such as military detection, geographic mapping, data acquisition, weather forecasting, etc. [1,2,3]. With the rapid development of aerial remote sensor technology, flight altitudes have reached tens of kilometers, and flight speeds have reached several Mach numbers. In this type of high-altitude and ultra-high speed flight condition, a complex air flow field is formed around aircraft, which puts forward more stringent requirements for the image quality of an aerial remote sensor [4]. The optical window is used to maintain the aerodynamic shape of the aircraft, provide an optical path for the internal photoelectric load, and isolate the external environment, and is directly exposed to the complex flight environment during aerial photography. The friction between a window glass and the atmosphere results in a significant amount of thermal load, which leads to additional stress and morphological changes on the surface of the window, and its mechanical and optical properties are seriously affected. Therefore, the imaging performance of the optical window is one of the key technologies restricting the application of a photoelectric load on hypersonic aerial remote sensors [5,6]. In order to meet the image quality requirements of aerial remote sensors under hypersonic flight conditions, it is necessary to rationally design the flexible supporting microstructure of the optical window to reduce the deformation of the window glass and to ensure the resolution and imaging quality of the remote sensor [7].

Researchers have conducted extensive studies on the structural design and imaging performance of the optical window. Aiming at the optical window of a large aperture ground-based telescope in the working temperature range of −35–40 °C, Q.P. Zhang et al. proposed an auxiliary support form of an intermediate ring belt based on an ordinary ring belt support, which could effectively improve the support efficiency, reduce the thickness and quality of a window, and reduce the window’s absorption rate of incident light [8]. M.L. Xu analyzed the optical window structure components of a space camera, and used the sealing gasket and the sealant layer (the applicable temperature range of the sealant material was from −65 to 250 °C) as the intermediate support structure of the window glass to improve the airtightness of the entire window assembly [9]. On the basis of subsonic flight conditions, Y.W. Li carried out an optical-mechanical-thermal integrated analysis of the optical window of an aerial remote sensor. The window support structure used a non-metallic rubber ring (the applicable temperature range of the rubber ring was from −100 to 300 °C), which reduced the deformation of the window glass and met the imaging requirements of the remote sensor [10]. According to the aerodynamic thermal effect and aerodynamic thermal radiation effect of optical window, L.Q. Dong et al. used the finite element analysis method to analyze the thermo-optic effect, elasto-optical effect, and thermal deformation of the optical window, and concluded that a non-uniform temperature gradient had a significant influence on the transmission optical path difference of the window, and the imaging quality of the optical system was reduced [11]. R. Zhang conducted a steady-state and transient simulation analysis of the optical window of a space solar telescope, and the temperature variation of the window component was ensured within a normal range by the thermal compensation method of electric heating [12]. Y.P. Zhang studied the influence of the optical path difference of deformed optical window in an aerodynamic thermal environment on the light transmission, calculated and analyzed the temperature field and stress field of the window, and discussed the effect of thermal load and atmospheric pressure on the transmission optical path difference of the window [13]. In addition, Y.L. Ding and others also performed an optimization analysis and verification on the parameters of optical window components, including materials, support forms, etc., and solved the technical problems faced by optical window applications with flying speeds below Mach 3 [14].

It can be seen that although researchers have carried out extensive studies on the imaging performance analysis and support structure design of optical window, they have mainly focused on the ranges of subsonic and supersonic and the flexible support structure based on non-metallic materials was used in the optical window, and few studies have analyzed the supporting structure of the optical window of a hypersonic remote sensor [15,16,17]. In this study, the optical window studied was mounted on a hypersonic aerial remote sensor with a flying speed of Mach 6 and a flying height of 20 km. The temperature of the outer surface of the optical window reached 800 °C due to aerodynamic thermal effects during operation. Under ultra-high temperature conditions, non-metallic super-elastic materials may soften, melt, and thermally oxidize, which may cause material performance failure. Therefore, the non-metallic flexible support structure and related technologies are no longer applicable. Therefore, it is necessary to select high-temperature resistant metal materials as the intermediate flexible support structure to reduce the complex thermal stress of the window glass. A honeycomb sandwich structure is a special form of laminated composite materials, which achieves the structural advantages of the entire system by using the performance characteristics of each component. A sandwich structure has outstanding characteristics such as light weight, high specific strength, adjustable in-plane stiffness, and stable high temperature performance. It has been widely used in main load-bearing structural parts such as wings, fuselage, and tail, but it is rarely used in flexible support structures. The semicircular ring-shaped honeycomb sandwich structure is a typical transverse-isotropic material, which has low in-plane rigidity, so that it can obtain greater elastic deformation, and also has good performance of vibration and noise reduction, heat insulation, and sound insulation [18,19].

The purpose of this study is to solve the problem that the thermal stress cannot be released due to the mismatch of the linear expansion coefficient of the visible optical window assembly of a hypersonic aerial remote sensor, which leads to the degradation of the imaging quality. A scheme of adding a high-temperature elastic alloy double-layer semicircular honeycomb sandwich flexible support microstructure between a window glass and a frame is proposed to reduce the influence of complex stress on the surface accuracy of the optical window and ensure the image quality of the remote sensor. The remainder of this paper is organized as follows: In Section 2, we establish the semicircular honeycomb sandwich flexible support structure and theoretically derive the equivalent model of elastic parameters of the semicircular honeycomb structure; in Section 3, we use static knowledge, dynamic modal analysis, and finite element simulation methods to verify the correctness of the equivalent model of the flexible support structure; in Section 4, we carry out a thermal-mechanical coupling simulation analysis on the overall components of the optical window, and the window surface deformation data are obtained and imported into the optical software by Zernike polynomial fitting, and the wave aberration and MTF value are used to evaluate the effectiveness of the semicircle honeycomb flexible support structure on the attenuation of the additional thermal stress of the window; finally, in Section 5, we analyze the data and summarize the work of this study.

## 2. Theoretical Model Analysis

A circular honeycomb sandwich structure has a flexible design and versatility. In order to accurately describe a circular honeycomb sandwich structure, especially a semicircular sandwich layer, a large number of elements must be divided into a finite element model. Therefore, even a very simple model requires a lot of time to solve. According to this reason, the theory of equivalence emerges, whose main goal is to make the complex sandwich structure equivalent to a transverse-isotropic plate, so that the whole circular honeycomb sandwich structure can be regarded as a layered material [20,21].

**Figure 1:**
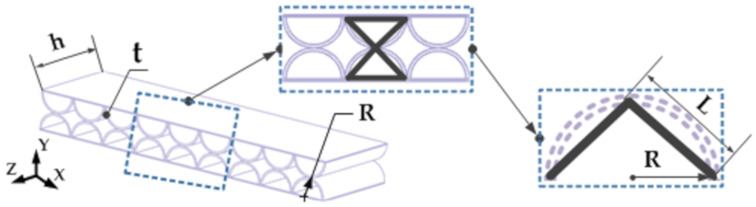
Double-layer semicircular honeycomb sandwich support structure and analysis unit. (*R*, *t*, *h*, respectively, represent the radius, wall thickness, and height of the circular honeycomb sandwich core).

The double-layer semicircular honeycomb sandwich support structure is shown in Figure 1. The double-layer semicircular cell elements are evenly distributed and externally tangent to each other, and the cross-section coincides with the surface of the two panels. The macroscopic elastic properties of the honeycomb structure are obtained from the properties of the smallest repeating unit in the honeycomb model. An analysis unit from the double-layer honeycomb structure is taken as the research object. According to the contact characteristics of the semicircular cell of the analysis unit, the contact points are connected by straight lines to form a regular triangular shape equivalent model. Therefore, assuming that the flexible support structure of the double-layer semicircular honeycomb sandwich layer can be enclosed by such right isosceles triangle cells, and the force form and direction of contact between semicircular cells are approximately equal to the force characteristics of triangular cells. According to the geometric relationship of the analysis unit model, we can get the ratio (relative density m) of the density $\rho_{s}$ of the honeycomb sandwich of the equivalent model and the density $\rho_{k}$ of the honeycomb wall material as follows:

(1) $$m=\frac{\rho_{s}}{\rho_{k}}=\frac{\pi }{4}\cdot \frac{t}{R}\cdot \left(2-\frac{t}{R}\right)$$

### 2.1. In-Plane Equivalent Elastic Modulus and Poisson’s Ratio

**Figure 2:**
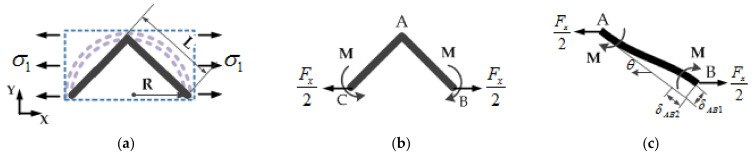
Stress situation of equivalent unit subjected to stress in the *X* direction: (**a**) The stress of the whole equivalent element; (**b**) the force distribution of equivalent cell walls; (**c**) the force on one cell wall of the equivalent element.

Firstly, the Cartesian coordinate system is established as shown in the figure. When the cell element of the honeycomb sandwich layer is subjected to the *X*-direction unidirectional plane stress, $\sigma_{1}$, the equivalent force of the triangle analysis unit is shown in Figure 2. We can obtain the tensile force, $F_{x}$, of the unit as:

(2) $$F_{x}=\sigma_{1}\cdot h\cdot 2R$$

The cell wall of *AB* is regarded as a flexible beam, combined with the deformation coordination condition that the rotation angle at point *B* is zero, and according to the relevant knowledge of mechanics, we obtain:

(3) $$\theta_{\frac{F_{x}}{2}B}=\frac{\frac{F_{x}}{2}\cdot \sin\left(\frac{\pi }{4}\right)\cdot L^{2}}{2E_{k}I},\;\theta_{MB}=\frac{M\cdot L}{E_{k}I}$$

where $I=\frac{ht^{3}}{12}$ is the moment of inertia of the section, $E_{k}I$ is the bending stiffness, and $E_{k}$ is the elastic modulus of the honeycomb cell material.

Because $\theta_{\frac{F_{x}}{2}B}=\theta_{MB}$, we can obtain:

(4) $$M=\frac{\sqrt{2}F_{x}L}{8}$$

The deflection of the *AB* cell wall perpendicular to the direction of *AB* caused by force $\frac{F_{x}}{2}$ and bending moment *M* is as follows:

(5) $$\delta_{AB1}=\frac{ML^{2}}{2E_{k}I}-\frac{\frac{F_{x}}{2}\sin\left(\frac{\pi }{4}\right)L^{3}}{3E_{k}I}=\frac{\sqrt{2}F_{x}L^{3}}{4E_{k}ht^{3}}$$

The axial deformation of the *AB* cell wall is as follows:

(6) $$\delta_{AB2}=\frac{\frac{F_{x}}{2}\cdot \cos\left(\frac{\pi }{4}\right)\cdot L}{E_{k}ht}=\frac{\sqrt{2}F_{x}L}{4E_{k}ht}$$

In this case, the effective strain in the *X* and *Y* directions becomes:

(7) $$\varepsilon_{1}=\frac{2\left(\delta_{AB1}\sin\theta +\delta_{AB2}\cos\theta \right)}{2L\cos\theta }=\frac{\sqrt{2}F_{x}L^{2}}{4E_{k}ht^{3}}\left(1+\frac{t^{2}}{L^{2}}\right)$$

(8) $$\varepsilon_{2}=\frac{\left(\delta_{AB2}\sin\theta -\delta_{AB1}\cos\theta \right)}{L\sin\theta }=-\frac{\sqrt{2}F_{x}L^{2}}{4E_{k}ht^{3}}\left(1-\frac{t^{2}}{L^{2}}\right)$$

Then, the equivalent Poisson ($\mu_{1}$) ratio and the equivalent in-plane elastic modulus ($E_{cx}$) in the *X* direction are:

(9) $$\mu_{1}=-\frac{\varepsilon_{2}}{\varepsilon_{1}}=\frac{1-t^{2}/L^{2}}{1+t^{2}/L^{2}}=\frac{1-t^{2}/2R^{2}}{1+t^{2}/2R^{2}}$$

(10) $$E_{cx}=\frac{\sigma_{1}}{\varepsilon_{1}}=\frac{\sqrt{2}E_{k}t^{3}}{RL^{2}\left(1+t^{2}/L^{2}\right)}=\frac{\sqrt{2}}{2}\cdot E_{k}\cdot {\left(\frac{t}{R}\right)}^{3}\cdot \frac{1}{1+t^{2}/2R^{2}}$$

**Figure 3:**
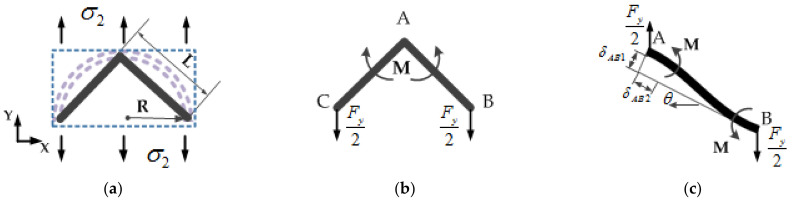
Stress situation of equivalent unit subjected to stress in the *Y* direction: (**a**) The stress of the whole equivalent element; (**b**) the force distribution of equivalent cell walls; (**c**) the force on one cell wall of the equivalent element.

Similarly, the equivalent Poisson ($\mu_{2}$) ratio and the equivalent in-plane elastic modulus ($E_{cy}$) in the *Y* direction can be derived. The analysis method is similar to the above, as shown in Figure 3.

Then, the equivalent Poisson ($\mu_{2}$) ratio and the equivalent in-plane elastic modulus ($E_{cy}$) in the *Y* direction are:

(11) $$\mu_{2}=-\frac{\varepsilon_{1}}{\varepsilon_{2}}=\frac{1-t^{2}/L^{2}}{1+t^{2}/L^{2}}=\frac{1-t^{2}/2R^{2}}{1+t^{2}/2R^{2}}$$

(12) $$E_{cy}=\frac{\sigma_{2}}{\varepsilon_{2}}=\frac{\sqrt{2}E_{k}t^{3}}{RL^{2}\left(1+t^{2}/L^{2}\right)}=\frac{\sqrt{2}}{2}\cdot E_{k}\cdot {\left(\frac{t}{R}\right)}^{3}\cdot \frac{1}{1+t^{2}/2R^{2}}$$

### 2.2. In-Plane Equivalent Shear Modulus

**Figure 4:**
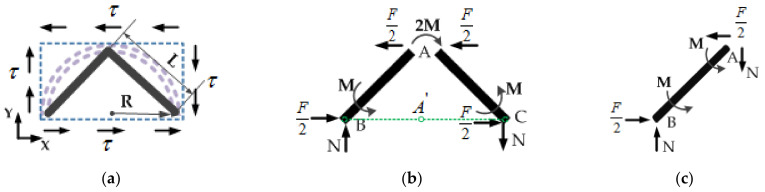
When subjected to shear stress, the force of equivalent element: (**a**) The stress of the whole equivalent element; (**b**) the force distribution of equivalent cell walls; (**c**) the force on one cell wall of the equivalent element.

When the analysis unit is subjected to shear stress, τ, the force analysis of the equivalent analysis element model is shown in Figure 4. In order to satisfy the force balance of each node, the following assumptions are made: (1) Assume that there is no relative displacement among the three nodes *A*, *B*, and *C*. (2) The corners of all nodes are equal. (3) The shear deformation of each side in the model is produced by its own rotation and bending deformation.

First, take the moment of point A′ on the *BC* line which is vertically projected by node A in Figure 4b, then $\sum M_{A^{\prime }}=0$, we can obtain:

(13) $$FL\sin\left(\frac{\pi }{4}\right)-2NL\cos\left(\frac{\pi }{4}\right)=0$$

It is concluded that:

(14) N=F2

Take the moment of node *A* in Figure 4c, then $\sum M_{A}=0$, we can obtain:

(15) $$2M+\frac{F}{2}L\cdot \sin\left(\frac{\pi }{4}\right)-N\cdot L\cdot \cos\left(\frac{\pi }{4}\right)=0$$

The conclusion is:

(16) M=0

Therefore, the shear deformation of each side in the equivalent analysis element is only the bending deformation, and the deflection $\delta_{AB}$ of the *AB* cell wall caused by the force $\frac{F}{2}$ is:

(17) $$\delta_{AB}=\frac{F}{2}\cdot \sin\left(\frac{\pi }{4}\right)\cdot \frac{L^{3}}{3EI}$$

Then, the shear strain $\gamma_{xy}$ and shear stress τ are:

(18) $$\gamma_{xy}=\frac{\delta_{AB}}{L}=\frac{\frac{F}{2}\cdot \sin\left(\frac{\pi }{4}\right)\cdot \frac{L^{3}}{3E_{k}I}}{L}=\frac{\sqrt{2}FL^{2}}{12E_{k}I}$$

(19) $$\tau =\frac{F}{2hL\cdot \cos\left(\frac{\pi }{4}\right)}=\frac{F}{\sqrt{2}hL}$$

According to the shear of Hooke’s law, the above formulas are used, and the shear modulus $G_{xy}$ in the semicircular honeycomb surface can be obtained as:

(20) $$G_{xy}=\frac{\tau }{\gamma_{xy}}=\frac{\frac{F}{\sqrt{2}hL}}{\frac{\sqrt{2}FL^{2}}{12E_{k}I}}=\frac{6E_{k}I}{hL^{3}}=\frac{\sqrt{2}}{8}\cdot E_{k}\cdot {\left(\frac{t}{R}\right)}^{3}$$

According to the above derivation, $E_{cx}=E_{cy}=E$, $\mu_{1}=\mu_{2}=\mu$, and satisfy $G_{xy}=\frac{E}{2\left(1+\mu \right)}$. Therefore, it is verified that the elastic constants of the semicircular honeycomb sandwich in the two main directions mentioned above are the same, and the ideal semicircular double-layer honeycomb structure is laterally anisotropic macroscopically, and isotropic in the *X*-*Y* plane. For the Cartesian coordinate system, the macroscopic stress–strain relationship of a double-layer semicircular honeycomb is as follows:

(21) $$\left[\begin{array}{c} \sigma_{x}\\ \sigma_{y}\\ \tau_{xy} \end{array}\right]=\left[\begin{array}{ccc} \frac{E_{cx}}{1-\mu_{1}\mu_{2}} & \frac{\mu_{1}E_{cy}}{1-\mu_{1}\mu_{2}} & 0\\ \frac{\mu_{2}E_{cx}}{1-\mu_{1}\mu_{2}} & \frac{E_{cy}}{1-\mu_{1}\mu_{2}} & 0\\ 0 & 0 & G_{xy} \end{array}\right]\left[\begin{array}{c} \varepsilon_{1}\\ \varepsilon_{2}\\ \gamma_{xy} \end{array}\right]$$

## 3. Finite Element Analysis and Verification of Equivalent Mode

**Figure 5:**
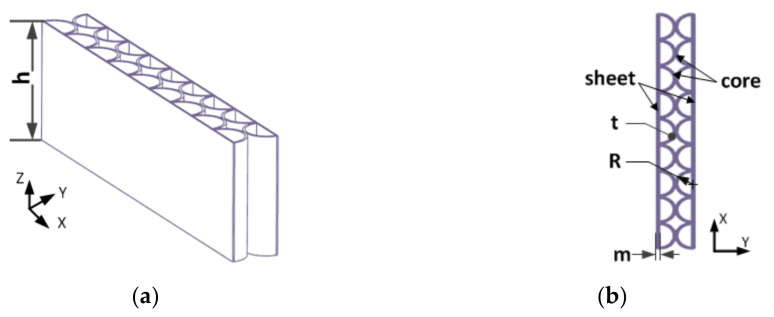
The double-layer semicircular honeycomb flexible support structure: (**a**) Isometric view; (**b**) top view.

Assuming that the double-layer semicircular honeycomb flexible support structure is as shown in Figure 5, the thin plates on both sides and the circular honeycomb core are made of nickel-based high temperature elastic alloy material (GH4099), which has excellent high temperature durability and high temperature creep performance; the physical properties are shown in Table 1. The thickness of the thin plate is *m*, the wall thickness of the semicircular honeycomb core is *t*, the radius of the circular honeycomb is *R*, and the height is *h* [22].

In order to verify the correctness and validity of the analytical expression of the equivalent model of the double-layer semicircular cell, we used the commercial finite element software ABAQUS to carry out the finite element analysis. In the mechanical simulation analysis of a honeycomb structure, it is generally considered that the fine modeling method can accurately characterize the structural characteristics and actual deformation state of a honeycomb plate. Therefore, we used the method of comparing the fine modeling with the equivalent modeling to verify the accuracy of the equivalent model of a double-layer semicircular honeycomb. The double-layer semicircular honeycomb sandwich layer is equivalent to the transverse anisotropic sandwich panel of the corresponding size. Six groups of different relative thicknesses (the ratio of the wall thickness to the radius of the honeycomb core layer) were used as data for the comparative analysis. According to the equivalent formula of the honeycomb core derived in the previous section, the physical properties of the intermediate layer can be obtained as shown in Table 2. It can be seen from Table 2 that as the relative thickness decreases, the in-plane elastic modulus and Poisson’s ratio of the equivalent model both decrease.

**Figure 6:**
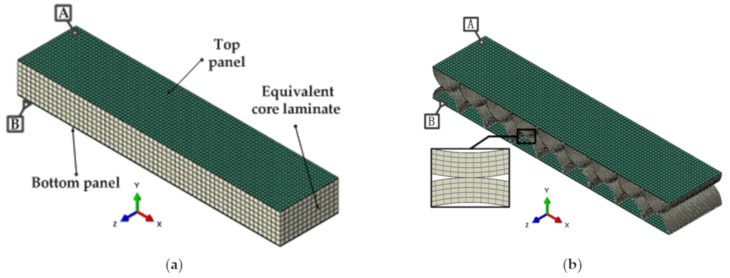
Finite element model of honeycomb sandwich structure: (**a**) Equivalent modeling method; (**b**) fine modeling method.

In the equivalent model, the coupling between the equivalent core plate and the upper and lower panels adopts the classic laminate theory. The faceplate and the core layer both adopt C3D8R (eight-node linear hexahedral element) three-dimensional stress solid element, and the core layer is given equivalent mechanical parameters and material properties. The finite element analysis model is shown in Figure 6a. In the fine modeling, the three-dimensional stress solid element (C3D8R) is also used for the panel and honeycomb thin wall, and the connection methods are all tie connections. The finite element analysis model is shown in Figure 6b.

### 3.1. Static Deformation Analysis

According to the stress environment of the honeycomb support structure in the actual window components, six degrees of freedom of all nodes on the bottom surface of the honeycomb panel at the *B* end were constrained in the static analysis. Tie constraints were used to connect the rigid body instigators on the upper surface of the *A* end honeycomb plate, and the in-plane shear load *Px* (force along the *X*-axis) and in-plane pressure load *Py* (force along the *Y*-axis) were uniformly applied, respectively. When using different modeling methods, the displacements of honeycomb panels under various static working conditions can be calculated, as shown in Table 3. Among them, in the *Px* and *Py* working conditions, the displacement is the average displacement component of all nodes on the lower surface of the *A* end honeycomb panel along the direction of force loading.

According to the data in Table 3, compared with the fine modeling method, the displacement error of the proposed equivalent modeling method is less than 2% under each working condition, and the maximum displacement error is only 1.88%, which indicates that the equivalent method has high accuracy in static analysis, and further verifies that the analytical method and the finite element method have a high degree of consistency in calculating equivalent parameters. A change in the relative thickness has an obvious influence on the elastic parameters of the honeycomb structure, and with a decrease in the relative thickness, the overall stiffness of the honeycomb structure is weakened, and the flexibility is enhanced. At the same time, it is also noticed that the calculated data of the equivalent model under each working condition are larger than the result of the fine model, which indicates that the stiffness of the model is weakened to a certain extent during the equivalence process.

### 3.2. Dynamic Modal Analysis

In order to verify whether the equivalent modeling method proposed in this study can accurately reflect the dynamic characteristics of the honeycomb sandwich, the modal analysis of the fine modeling method and the equivalent modeling method are carried out [23]. Table 4 shows the first-order modal analysis results of fine modeling and equivalent modeling of six groups of different relative thicknesses.

The data in Table 4 show that the maximum difference in the first-order fundamental frequency of the six groups of fine modeling and equivalent modeling with different relative thicknesses is 4.01%. From the deformation cloud chart, it can be seen that the mode shapes of the fine modeling method and the equivalent modeling method are basically the same, which indicates that the equivalent modeling method derived in this study meets the accuracy requirements of the analysis in the dynamic analysis of the semicircular honeycomb sandwich structure, and the modal characteristics of the sandwich structure can be well preserved. At the same time, it can be seen that using the equivalent modeling method proposed in this study, the calculated fundamental frequency data of the first-order modal are slightly smaller than those of the fine modeling method, indicating that the equivalent modeling method deduced in this study weakens the stiffness of the semicircular honeycomb sandwich structure to a certain extent.

In addition, in the simulation analysis, when using the equivalent modeling method proposed in this study to analyze the statics and the first mode, the calculation process (CPU time) is only 2 s, which is much less than the 10 s of the fine modeling method. It can be seen that the equivalent modeling method proposed in this study has significant advantages in computational efficiency.

## 4. Analysis of Window Component and Verification of the Flexible Support Structure

In the actual working process of the hypersonic remote sensor, the strong interaction between the outer surface of the optical window and the air flow generates aerodynamic heat, and the temperature of the outer surface of the window increases sharply, resulting in the nonlinear temperature gradient of the axial and radial direction of the window glass, causing deformation of the window glass and the supporting elements [24,25]. Due to the mismatch of the thermal expansion coefficients, the window produces contact stress, which directly leads to a change in the internal and external surface shapes of the window. The double-layer semicircular honeycomb sandwich flexible support structure can effectively reduce the contact thermal stress, and ensure the wave aberration accuracy of the window transmission optical path difference and the optical performance of the remote sensor system.

In this section, based on the ABAQUS finite element software, the thermal-mechanical coupling simulation analysis of the window assembly with flexible support structure is performed, and the deformation data of the inner and outer surfaces of the window are obtained. A Zernike polynomial is used to fit the data as the base function. The Zernike polynomial coefficients are substituted into the optical analysis software ZEMAX to analyze the image quality, and the wave aberration of the transmitted optical path difference and the MTF value of the optical system are used to evaluate the effectiveness of the double-layer semicircular honeycomb flexible support structure to attenuate the additional thermal stress of the window.

**Figure 7:**
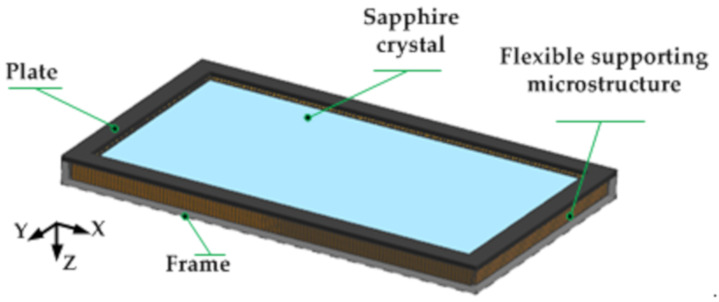
Schematic diagram of optical window assembly.

The actual optical window component adopts a single-layer window structure with the advantages of a simple structure, small size, and light weight, as shown in Figure 7. The window glass is made of sapphire material with low density, high elastic modulus, and high thermal conductivity, surrounded by a high temperature elastic alloy honeycomb sandwich flexible support structure, and finally the window glass and supporting structure are fixed on the C/SiC composite frame with a pressing plate [26,27].

### 4.1. Optical-Mechanical Thermal Integration Analysis

**Figure 8:**
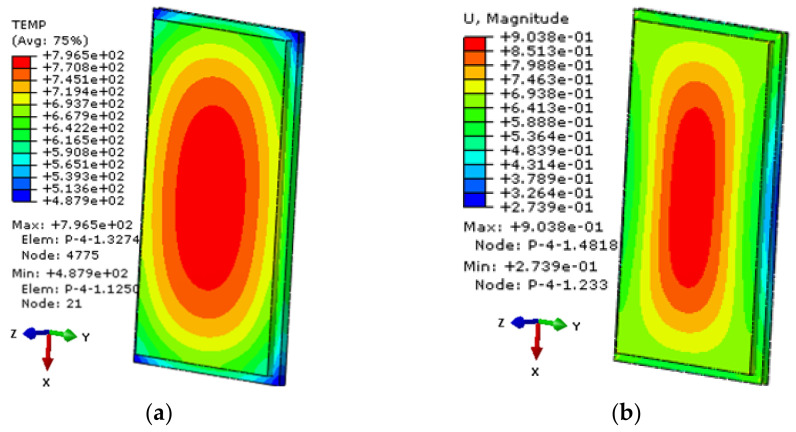
The thermal-structural coupled analysis of the window components: (**a**) Cloud diagram of the window glass temperature distribution; (**b**) deformation displacement cloud diagram of the window glass (the direction of window light is *Z* direction).

The window components with equivalent flexible support structure are imported into the ABAQUS software, and the heat flux on the outer surface of the window glass obtained by aerodynamic thermal analysis is taken as the working condition of thermal analysis. The window temperature field distribution cloud diagram and the thermal deformation displacement cloud diagram when the temperature difference between the inside and outside of the window glass is maximum are obtained, as shown in Figure 8. According to Figure 8a, the maximum temperature of the outer surface of the window glass will reach 800 °C, and the middle temperature is higher than the edge temperature. As can be seen from Figure 8b, the deformation in the middle of the window glass is greater than that at the edge, and the window changes from a plane to a curved surface and bulges outward.

**Figure 9:**
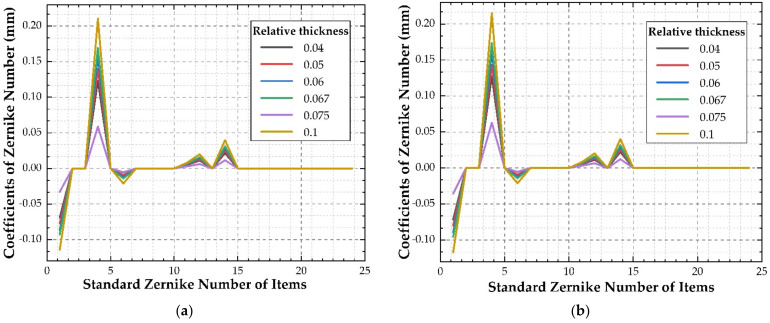
Zernike coefficients of the first 24 items of the inner and outer surface shape of the window glass with honeycomb structure of different relative thicknesses: (**a**) Inner surface; (**b**) outer surface.

**Figure 10:**
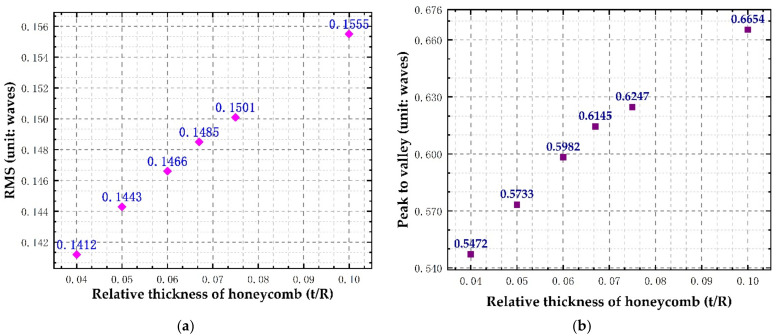
Wave aberration of the transmitted optical path difference of the window glass with honeycomb structure of different relative thicknesses: (**a**) RMS value of transmitted optical path difference; (**b**) PV value of transmitted optical path difference.

According to the simulation results of the window glass deformation, the wave aberration values of transmitted optical path difference of each simulation point are calculated, the Zernike polynomials on the inside and outside surfaces of the window glass are fitted by the least square method, the coefficients of the first 24 Zernike polynomials are obtained (as shown in Figure 9), and the rigid body displacement is separated (as shown in Table 5). The transmission optical path difference wave aberration values (PV, RMS) of the window glass caused by honeycomb flexible support structure with different relative thicknesses are calculated, as shown in Figure 10.

We can see that with a decrease in the relative thickness of the honeycomb support structure, the aberration of the transmitted optical path of the window glass gradually decreases. Due to the large temperature difference between the inner and outer surfaces of the window glass, the displacement of the rigid body in the Z direction is relatively large, and the displacement of the rigid body in the other directions is relatively small. When the relative thickness is 0.100, the transmitted optical path difference RMS = 0.1555 λ, which is less than λ/6 (λ = 632.8 nm), which meets the design requirements of the optical system, indicating that the flexible support structure has a good effect on the thermal stress attenuation of the window.

### 4.2. Optical Performance Evaluation

**Figure 11:**
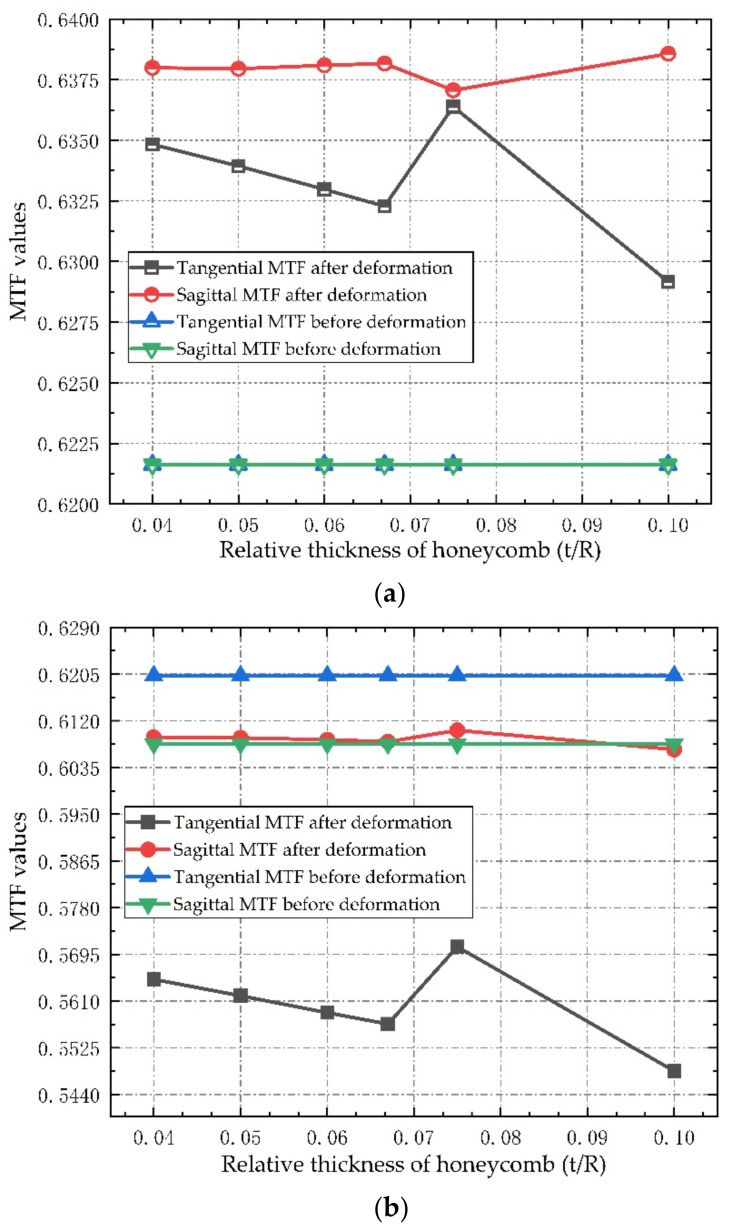

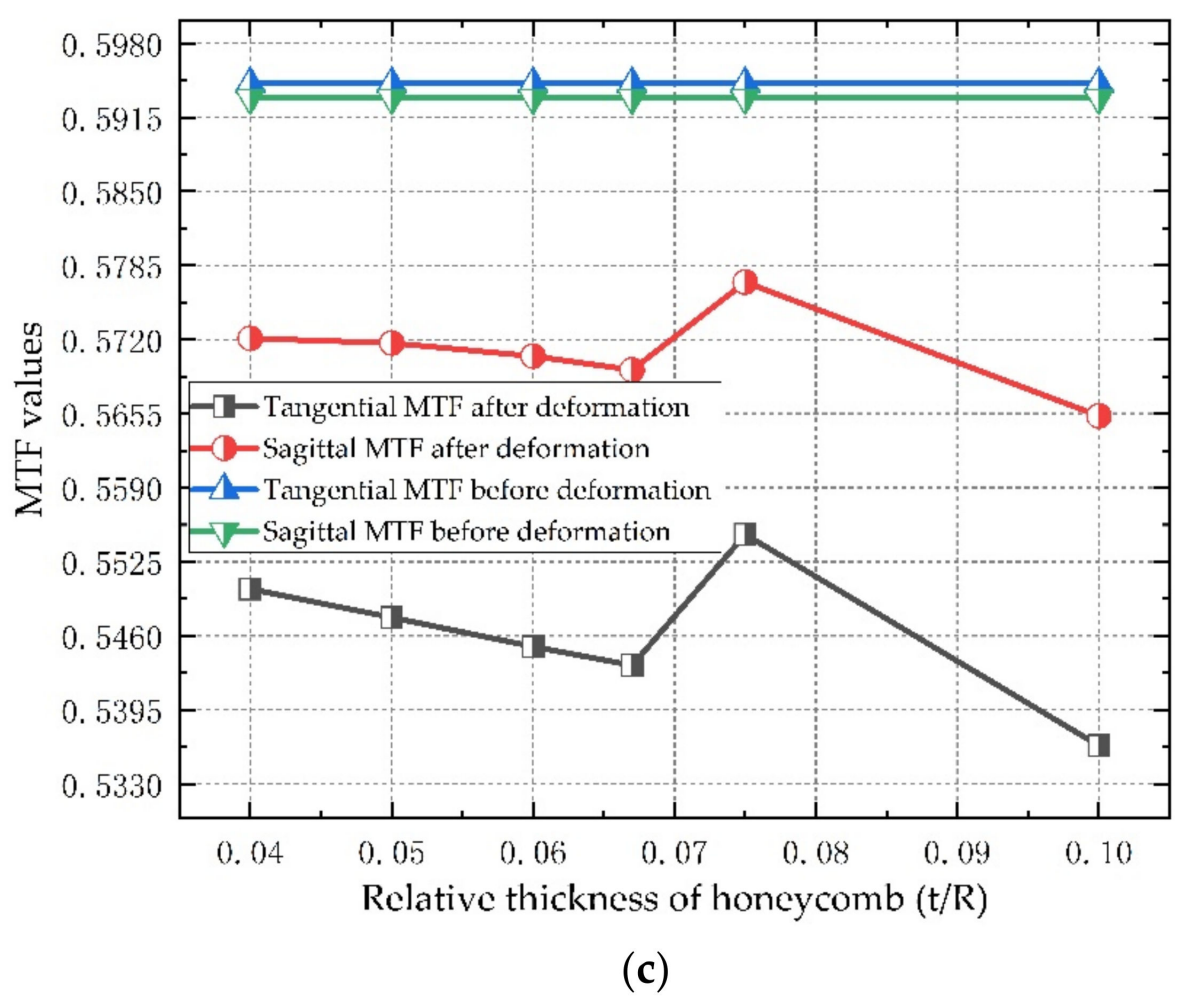
MTF values of the three fields of view before and after the additional deformation window of the optical system at a frequency of 63 cycle/mm: (**a**) Axial field of view; (**b**) 0.7 field of view; (**c**) full field of view.

The Zernike coefficient and rigid body displacement caused by thermal deformation of window glass are assigned to the optical system, and the influence of flexible support structures with different relative thicknesses on the imaging performance of optical window under thermal mechanical coupling is calculated. Figure 11 shows the MTF values of the three fields of view at a frequency of 63 cycle/mm before and after the introduction of the deformed window glass in the optical system.

As can be seen from Figure 11, after the introduction of deformed window glass, the MTF values of the three fields of view slightly increase or decrease, but the range of change is very small, and the maximum decrease of MTF is less than 0.073, which meets the design requirements of the optical system. It is also noted that with a decrease in the relative thickness, the change of MTF value decreases gradually, indicating that the honeycomb flexible support structure has a good effect on the thermal stress release of window glass. According to the analysis of optical performance, the double-layer semicircular honeycomb flexible support structure has little influence on the deformation of the window glass, which can be approximated as a positive lens with very small focal power outside the cabin. After introducing it into the optical system, it causes the aberration of some fields of view to increase, but at the same time it may also compensate the aberration in some directions of other fields of view, so that the MTF value shows a slight change trend of increasing and decreasing in different fields of view after the introduction of deformed window glass. However, due to the small change value, the image quality of the optical system does not change significantly after focusing, which meets the image quality requirements of the remote sensor.

## 5. Discussion

**Figure 12:**
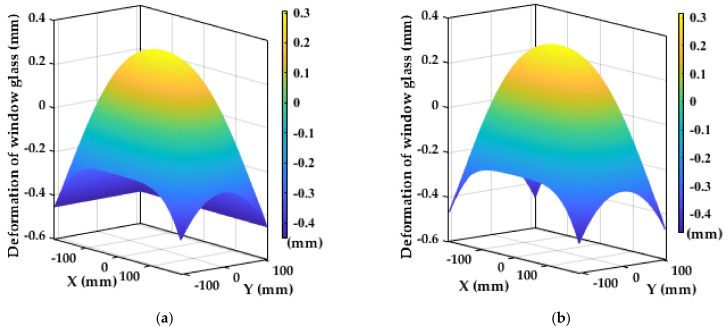
Standard Zernike phase fitting residual surface shape of the inner and outer surfaces after the window glass is deformed: (**a**) The inner surface; (**b**) the outer surface.

**Figure 13:**
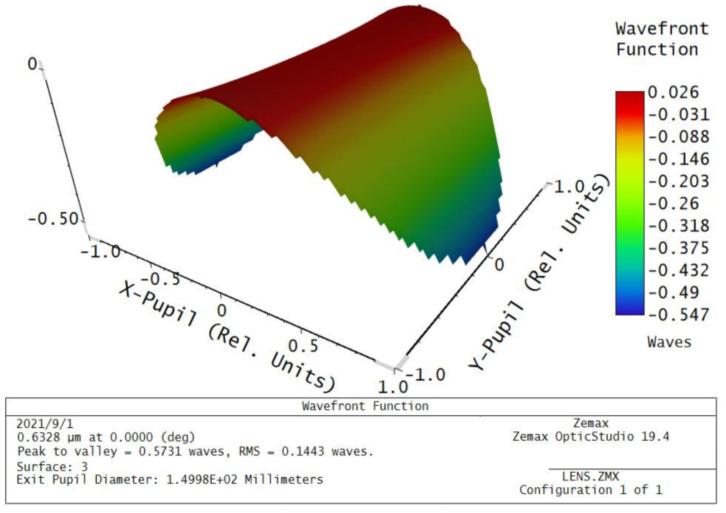
Residual surface shape of the transmitted optical path difference of window glass.

According to the analysis of the above six groups of honeycomb structures with different relative thicknesses, from the perspective of the static analysis, when the relative thickness of the honeycomb structure decreases, the elastic modulus of the honeycomb sandwich decreases, the flexibility increases, and the ability to attenuate the complex thermal stress of window glass becomes stronger. However, from the perspective of the dynamic analysis, the smaller the relative thickness of the honeycomb structure, the lower the density of the honeycomb core, which leads to a reduction in the stiffness of the supporting structure, insufficient bearing capacity, and a decrease in the natural frequency. In severe cases, the aerial remote sensor will not be able to image, and even destroys the window components. From the perspective of the imaging performance analysis, when the relative thickness of the honeycomb structure decreases, the smaller the influence of the support structure on the wave aberration of the transmission optical path difference of the window glass, the better the imaging quality of the remote sensor. Therefore, the honeycomb sandwich support structure needs to have good deformability to reduce the thermal stress of the window, and also needs to have a certain bearing capacity. According to the above simulation results, the vibration frequency of aircraft, and the overall size requirements of window components, we choose the size parameter of double-layer semicircular honeycomb with a relative thickness of 0.05 as the best flexible support structure. At this time, after the window glass is deformed, the standard Zernike fitting residual surface diagram of the inner and outer surfaces to remove the rigid body displacement is shown in Figure 12, and the wave aberration of the transmitted optical path difference of the window glass is shown in Figure 13. As can be seen from Figure 13, the temperature field and the stress field cause the transmitted optical path difference of the window glass to form a slope on four corners, and the optical path difference at the center of the window is less than that at the edge.

**Figure 14:**
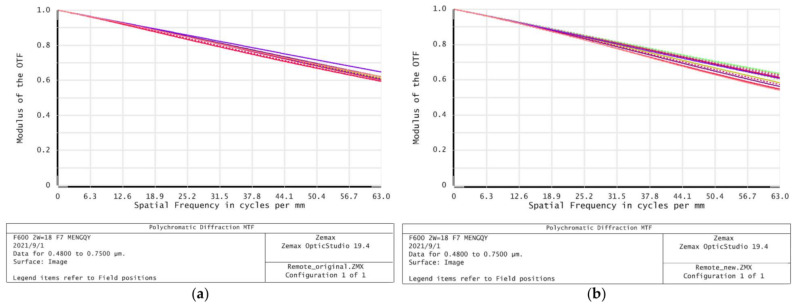
MTF curves of the optical system: (**a**) All fields without deformation; (**b**) all fields based on deformation effect.

**Figure 15:**
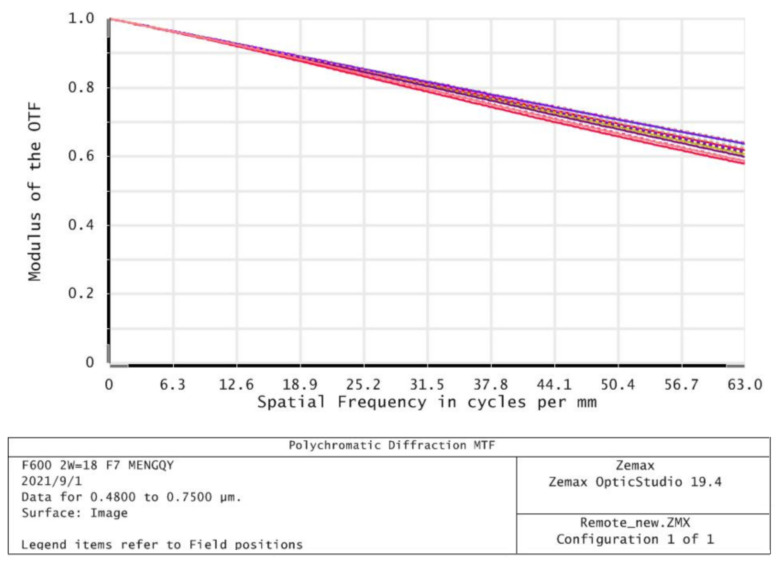
MTF curves of the optical system after focusing.

The spatial frequency of modulation transfer function (MTF) of the optical system before and after window glass deformation is 0.59 and 0.55 at 63 cycle/mm, respectively, as shown in Figure 14. The spatial frequency of the optical system modulation transfer function (MTF) of the deformed window glass after system focusing is 0.58 at 63 cycles/mm, as shown in Figure 15. Therefore, the image quality of the optical system before and after the introduction of deformable window glass has no obvious change after focusing, which meets the requirements of image quality.

## 6. Conclusions

Because the flight speed of aerospace vehicles has increased significantly, the imaging performance requirements of photoelectric payloads have also become higher and higher. Aiming at the optical window of a hypersonic remote sensor, in this study, a method is proposed for adding a double-layer semicircular honeycomb sandwich flexible support structure between a window glass and a frame, which solves the problem that the optical system is unable to image due to thermal deformation of the window glass under ultra-high temperature conditions. The following conclusions can be drawn from the analysis results:

(1) On the basis of the element cell model, the in-plane elastic parameters of the equivalent model of double-layer semicircular honeycomb are derived analytically, and verified numerically by finite element analysis. From the perspective of statics, comparing the relative thickness of six groups of different honeycomb sandwiches, the relative error of the static deformation of the theoretical fine model and the equivalent model is less than 2%. From the perspective of dynamics, the relative errors of the first-order modal fundamental frequency of the fine model and the equivalent model are both less than 4.01%. The correctness of the theoretical expression of the equivalent model is verified. At the same time, it is pointed out that as the relative thickness decreases, the elastic modulus and Poisson’s ratio in the plane of the semicircular honeycomb sandwich are both reduced. The elastic modulus and Poisson’s ratio in the two main directions of the semicircular honeycomb sandwich are the same, that is, it is an isotropic plane in the *X*-*Y* plane.
(2) On the basis of the integrated analysis of the optical window components with flexible supported microstructure with honeycomb sandwich, the thermal deformation data of the inner and outer surface of the window glass were obtained according to the heat flux of the outer surface of the window as the thermal boundary condition. According to a Zernike polynomial, the least square method was used to fit the deformation data to determine the coefficient of the Zernike polynomial, which was substituted into the optical software ZEMAX to analyze the imaging performance. It is calculated that the wave aberration PV and RMS values of the transmitted optical path difference of the optical window caused by the honeycomb flexible support structure with different relative thicknesses are less than 0.665 λ and 0.156 λ (λ = 632.8 nm), which meets the system design requirements. The MTF values of each field of view of the optical system after the introduction of window glass deformation are greater than 0.55 (63 cycle/mm), and the decreases MTF values are less than 0.073, which meets the design requirements of the optical system. Finally, the flexible support structure with relative thickness of 0.05 is discussed, and the MTF value of each field of view of the deformed window glass after focusing by the optical system is greater than 0.58 (63 cycle/mm), and the image quality of the aerial remote sensor has no significant change, which meets the imaging requirements.

The design and analysis results show that the double-layer semicircular honeycomb sandwich flexible support microstructure proposed in this study, which is applied to the support structure design of a hypersonic remote sensor optical window, effectively reduces the thermal stress and deformation of window glass, ensures the imaging quality of photoelectric load, and provides a new solution for the support structure design of a hypersonic optical window.

## Figures and Tables

**Table 1:** Material parameters of GH4099 at 800 °C.

$\mathrm{Density}\;\rho_{k}\;(g/{\mathrm{cm}}^{3})$	$\mathrm{Elastic}\;\mathrm{Modulus}\;E_{k}\;(\mathrm{Gpa})$	$\mathrm{Poisson}’s\;\mathrm{Ratio}\;\mu_{k}$
8.47	178	0.33

**Table 2:** Equivalent physical properties of honeycomb core layers with different relative thicknesses.

Structural Parameters	tR	ρ (g/cm^3^)	$E_{1}\;(\mathrm{MPa})$	$G_{12}\;(\mathrm{MPa})$	$\mu_{12}$
*t* = 0.1 mm, *R* = 1.5 mm	0.067	0.8574	37.21	9.32	0.9956
*t* = 0.15 mm, *R* = 1.5 mm	0.100	1.2639	125.24	31.47	0.9900
*t* = 0.1 mm, *R* = 2 mm	0.050	0.6486	15.56	3.93	0.9975
*t* = 0.15 mm, *R* = 2 mm	0.075	0.9604	52.95	13.27	0.9944
*t* = 0.1 mm, *R* = 2.5 mm	0.040	0.5215	8.05	2.01	0.9984
*t* = 0.15 mm, *R* = 2.5 mm	0.060	0.7743	27.14	6.80	0.9964

**Table 3:** Deformation of a honeycomb sandwich structure under static conditions.

Working Condition of Value	Relative Thickness (*t*/*R*)	Displacement/mm		Relative Error/%
		Fine Modeling	Equivalent Modeling	
*Px* = 1000 N	0.100	2.342 × 10^−2^	2.381 × 10^−2^	1.67
	0.075	6.244 × 10^−2^	6.312 × 10^−2^	1.09
	0.067	1.026 × 10^−1^	1.038 × 10^−1^	1.17
	0.060	1.297 × 10^−1^	1.321 × 10^−1^	1.85
	0.050	2.103 × 10^−1^	2.135 × 10^−1^	1.52
	0.040	4.146 × 10^−1^	4.201 × 10^−1^	1.33
	0.033	7.285 × 10^−1^	7.369 × 10^−1^	1.15
*Py* = 1000 N	0.100	1.226 × 10^−2^	1.249 × 10^−2^	1.88
	0.075	3.928 × 10^−2^	4.001 × 10^−2^	1.86
	0.067	6.068 × 10^−2^	6.159 × 10^−2^	1.50
	0.060	8.579 × 10^−2^	8.681 × 10^−2^	1.19
	0.050	1.375 × 10^−1^	1.399 × 10^−1^	1.75
	0.040	2.791 × 10^−1^	2.838 × 10^−1^	1.68
	0.033	7.281 × 10^−1^	7.396 × 10^−1^	1.58

**Table 4:** First-order modal simulation results of fine modeling method and equivalent modeling method.

Relative Thickness (*t*/*R*)	Frequency/Hz		Relative Error/%	Modal Displacement Vector	
	Fine Modeling	Equivalent Modeling		Fine Modeling	Equivalent Modeling
0.100	6191.43	6058.19	2.15	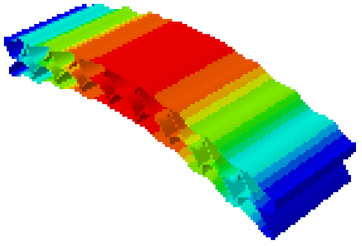	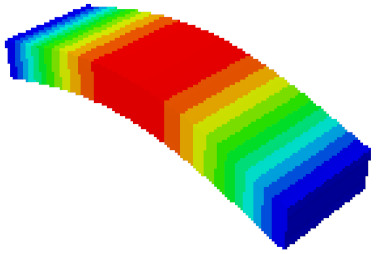
0.075	4513.19	4391.43	2.70	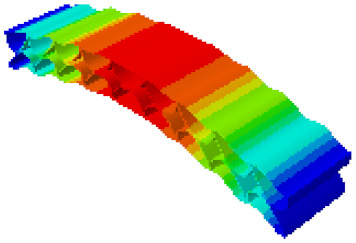	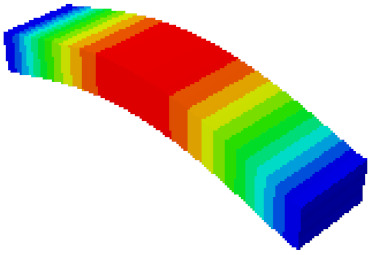
0.0667	3386.20	3260.25	3.72	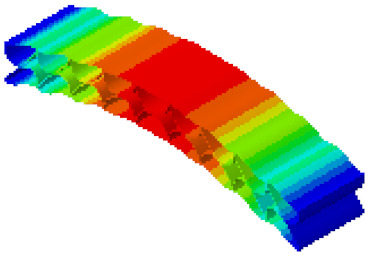	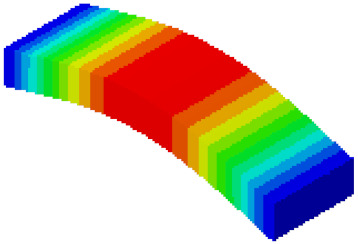
0.060	2404.19	2358.03	1.92	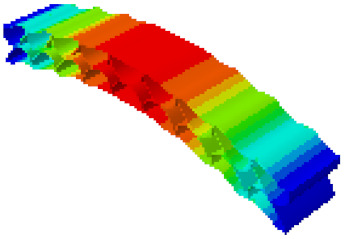	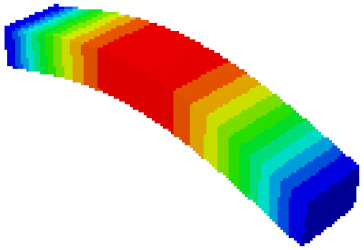
0.050	2113.96	2060.37	2.54	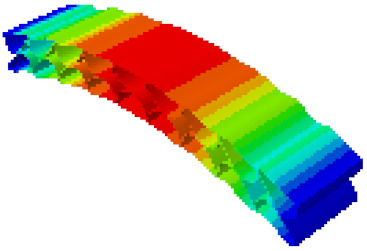	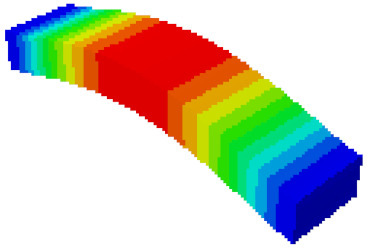
0.040	1502.17	1441.96	4.01	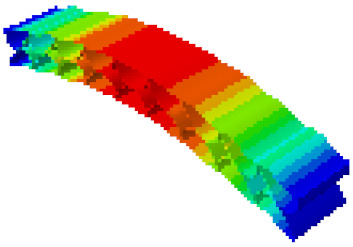	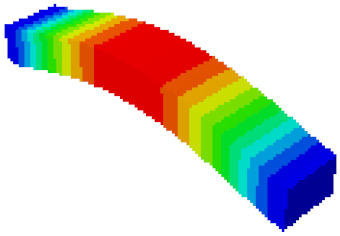

**Table 5:** Rigid body displacement of the window glass with different relative thicknesses of honeycomb structure.

t/R	Δ*x*/(mm)	Δ*y*/(mm)	Δ*z*/(mm)	$\theta_{x}$ /(°)	$\theta_{y}$ /(°)	$\theta_{z}$ /(°)
0.100	9.86185 × 10^−7^	1.79315 × 10^−7^	6.02882 × 10^−1^	7.95334 × 10^−8^	−6.93202 × 10^−8^	−8.20848 × 10^−9^
0.075	8.64046 × 10^−7^	1.37336 × 10^−7^	1.65878 × 10^−1^	1.52910 × 10^−8^	−9.39628 × 10^−9^	−1.35976 × 10^−8^
0.067	1.42106 × 10^−6^	1.44291 × 10^−7^	4.97727 × 10^−1^	7.33628 × 10^−8^	−8.91852 × 10^−8^	2.36528 × 10^−8^
0.060	1.56238 × 10^−6^	1.44926 × 10^−7^	4.66811 × 10^−1^	8.63635 × 10^−8^	−1.04217 × 10^−7^	3.21196 × 10^−8^
0.050	1.36385 × 10^−6^	1.75820 × 10^−7^	4.17189 × 10^−1^	8.58669 × 10^−8^	−1.06514 × 10^−7^	8.96065 × 10^−9^
0.040	1.16356 × 10^−6^	3.33384 × 10^−7^	3.72572 × 10^−1^	1.48376 × 10^−7^	−1.21973 × 10^−7^	−6.45510 × 10^−8^

## Data Availability

The data and results used to support the research in this article can be obtained from the corresponding author.
